# Clinical characteristics and prognostic analysis of patients with type 2 diabetic kidney disease and non-diabetic kidney disease

**DOI:** 10.3389/fendo.2025.1493521

**Published:** 2025-03-07

**Authors:** Can Yu, Wen Shi, Enhui Chen, Yang Qiu, Li Gao, Hansheng Fang, Jun Ni, Dongrong Yu, De Jin

**Affiliations:** Department of Nephrology, Hangzhou TCM Hospital of Zhejiang Chinese Medical University (HangZhou Hospital of Traditional Chinese Medicine), Hangzhou, China

**Keywords:** diabetes, diabetic kidney disease (DKD), diabetic retinopathy (DR), non-diabetic kidney disease (NDKD), prognostic analysis

## Abstract

**Background:**

In diabetic patients, non-diabetic kidney disease (NDKD) may occur independently or alongside diabetic kidney disease (DKD). This study explored the utility of kidney biopsy in type 2 diabetes mellitus (T2DM) patients and the predictability of diagnosing DKD combined with NDKD using clinical and laboratory data.

**Methods:**

This retrospective study examines medical records of T2DM patients who underwent percutaneous renal biopsy at Hangzhou TCM Hospital, Zhejiang Chinese Medical University, from 2012 to 2023. The patient’s demographic, clinical, blood test and pathological examination data were retrieved from their medical records. Multivariate regression analysis evaluated predictive factors for NDKD superimposed on DKD (DKD+NDKD).

**Results:**

A total of 285 patients were analyzed. The average age at the time of renal biopsy was 53.26 ± 10.55 years. The duration of diabetes was 93.19 ± 70.78 months. Of the patient population, 35.44% (101/285) were diagnosed with DKD alone, while 64.56% (184/285) had DKD+NDKD. Immunoglobulin A nephropathy was the most common pathological type in the DKD+NDKD group, accounting for 37.30% of the patients. Cystatin C [HR=2.688, 95% CI 1.035-6.879, P < 0.05] independently predicted the prognosis of patients with DKD+NDKD.

**Conclusions:**

These findings suggest that cystatin C plays a role in influencing the prognosis of patients with DKD + NDKD, indicating that NDKD patients might require distinct treatment strategies compared to those with DKD alone. However, further prospective clinical trials are needed to provide more clarity on the prognosis and outcomes of diabetic patients.

## 1 Background

The prevalence of diabetes has increased significantly over the past few decades. Diabetic kidney disease (DKD) is the primary complication, with diabetes-related chronic kidney disease (CKD) being more prevalent than CKD caused by chronic glomerular disease (1). The diagnosis of DKD primarily depends on proteinuria and renal function. Nevertheless, patients with diabetes may experience other forms of kidney disease beyond DKD (2). It is challenging to differentiate DKD from non-diabetic kidney disease (NDKD) by non-pathological examinations such as clinical manifestations and laboratory tests. Clinically, as part of CKD in patients with careful consideration of invasive risks and costs, and experience give clinicians follow DKD after clinical diagnosis (2). No further definite pathological diagnosis or pathological examination makes some NDKD delay treatment. Ultimately, it results in end-stage renal disease (ESRD). Renal biopsy is the sole dependable method for diagnosing renal disease in diabetic patients (3). Current guidelines lack consensus on the indications for renal biopsy in patients with T2DM and CKD. It is necessary to explore further the clinical manifestations and laboratory indicators that can indicate the occurrence of DKD+NDKD patients. These indicators can be used to determine whether there is the possibility of DKD+NDKD and whether it is necessary to perform a renal biopsy to confirm the diagnosis (4, 5). Early identification and adequate treatment of patients with diabetes mellitus complicated by chronic kidney disease, including those with DKD and those with DKD+NDKD, are crucial. According to the pathological characteristics, T2DM with kidney disease can be divided into DKD, diabetes with NDKD, and DKD+NDKD (6). Distinguishing between DKD and DKD+NDKD is challenging. This retrospective study analyzes the clinicopathological characteristics and prognosis of patients with T2DM complicated by renal disease (DKD and DKD with NDKD) to enhance clinical diagnosis and treatment decisions.

## 2 Method

This study was a retrospective analysis conducted on data from 285 T2DM patients who underwent kidney biopsies between 2012 and 2023 at the Department of Nephrology, Hangzhou Hospital of Traditional Chinese Medicine, Hangzhou, China. These data encompassed demographic information, clinical details, blood test outcomes, pathological findings, and survival results. This study’s T2DM diagnosis adheres to the China Diabetes Society criteria (7, 8). The inclusion criteria were 1) Chinese ethnicity and 2) CKD diagnosis; 3)T2DM at renal biopsy and no prior renal replacement therapy. The exclusion criteria included: 1) Type 1 diabetes and other diabetes types; 2) Uncorrectable bleeding tendency or severe hypertension; 3) Rapidly deteriorating renal function (4); Active kidney infection or acute kidney injury. All-cause death, doubling of serum creatinine, or renal replacement therapy were used as endpoint events. Patient demographics, clinical characteristics, and laboratory results were collected from patient files and electronic hospital records during kidney biopsy. Demographic and clinical data included gender, age, body mass index (BMI), duration of diabetes, glycated haemoglobin (HbA1c), serum albumin, total cholesterol, triglycerides, Low-Density Lipoprotein Cholesterol (LDL), 24-hour urinary protein, urinary albumin creatinine ratio, serum creatinine, blood urea nitrogen, blood uric acid, estimated glomerular filtration rate (eGFR by CKD-EPI), N-acetyl-beta-D-glucosaminidase (NAG), and Cystatin C. In addition, whether the patients had complications such as hypertension, diabetic retinopathy (DR), and cardiovascular and cerebrovascular diseases (CVD), whether they were treated with immunosuppressants (glucocorticoids or cytotoxic drugs, such as mycophenolate mofetil, cyclophosphamide, etc.), renin-angiotensin system blockers (ARB/ACEI), and tripterygium wilfordii polyglycosides tablets (TWP), and follow-up time and renal survival status were collected. Renal biopsy to perform all the procedures is unified. This study applied consistent renal biopsy criteria for all patients. Kidney biopsies were conducted with patient consent. For light microscopy: Hematoxylin and eosin (HE), periodic acid-Schiff (PAS), Masson’s trichrome (Masson), and periodic acid methenamine silver (PAMS). For immunofluorescence microscopy: IgG, IgM, IgA, C3 and C1q, Kappa and Lambda light chains, and Fibrinogen (9, 10). The pathological diagnosis and classification of DKD followed the criteria established by the Renal Pathology Society in 2010 (11). Based on renal biopsy pathology, patients were categorized into the isolated DKD group or the Mix group (DKD+NDKD).

### 2.1 Statistical analysis

Descriptive statistics were utilized to characterize the data, with normally distributed variables expressed as the mean ± standard deviation, median values for those with non-normal distributions, and frequencies (percentages) for categorical variables. To evaluate the impact of the treatment on continuous variables with a typical distribution, paired t-tests were applied. For comparisons across multiple groups, an analysis of variance (ANOVA) was conducted to determine the statistical significance of inter-group differences. In instances where the assumptions of ANOVA were not met, the Kruskal-Wallis test was employed to assess the significance of differences in mean values. Categorical variables were analyzed using either Pearson’s chi-square test or Fisher’s exact test, depending on the sample size and distribution. Statistical significance was set at the conventional threshold of P < 0.05. To mitigate the influence of confounding factors, propensity score matching (PSM) was implemented to equitably match subjects between the DKD and DKD+NDKD groups in a 1:1 ratio. A caliper value of 0.2 was applied to ensure balance in baseline characteristics. Potential confounders that significantly affected the study outcomes were identified through a systematic literature review and a stepwise logistic regression analysis.

## 3 Results

### 3.1 Study population characteristics

In this study, we conducted a comprehensive analysis of 285 patients who underwent renal biopsy for the diagnosis of T2DM, with the cohort comprising 208 males (73%) and 77 females (27%). The mean age at the time of biopsy was 53.26 ± 10.55 years, and the average duration of diabetes was 93.19 ± 70.78 months. Serum creatinine levels were recorded at 156.41 ± 130.57 µmol/L. The study population was categorized into two groups: DKDgroup, consisting of 101 cases, and the NDKD DKD+NDKD group, encompassing 184 cases. The DKD group included 68 males (68%), while the DKD+NDKD group had 140 male cases (76.1%). There was no significant difference in gender distribution between the two groups (P > 0.05). It is noteworthy that the DKD group exhibited a longer duration of diabetes (P < 0.05), a higher prevalence of diabetic retinopathy (P < 0.005), and a greater incidence of cardiovascular comorbidities (P < 0.05) compared to the DKD+NDKD group. Among those with DKD, 101 patients presented with proteinuria, with 46 cases of nephrotic-range proteinuria and 55 cases of mild-to-moderate proteinuria. Conversely, the DKD+NDKD group included 71 patients with nephrotic-range proteinuria and 113 with mild-to-moderate proteinuria. The prevalence of proteinuria was not significantly different between the groups, with the majority of cases falling into the mild-to-moderate category. Microalbuminuria, as indicated by the albumin-to-creatinine ratio (ACR), was significantly higher in the DKD group compared to the DKD+NDKD group (P < 0.05). Additionally, the DKD+NDKD group showed higher levels of BMI, serum albumin, alanine aminotransferase, and bilirubin compared to the DKD group (P < 0.05). No significant differences were observed in underlying conditions such as blood uric acid, NAG enzyme activity, anemia, hematuria, hypertension, and hyperthyroidism, except for cardiovascular disease and hyperlipidemia (P > 0.05). There were also no statistically significant differences between the two groups in terms of essential treatments, including hypoglycemic, lipid-lowering, antiplatelet, and anticoagulant medications (**Table 1**).

**Table 1:** Distribution of baseline parameters in pre-PSM patients in the DKD group and the DKD+NDKD group.

Parametric	DKD (n=101)	DKD+NDKD (n=184)	P
Age (years)	52.00 ± 10.37	53.47 ± 10.86	
Male [cases (%)]	68 (68)	140 (76.1)	
BMI (kg/m^2^)	24.51 ± 3.14	25.53 ± 2.73	<0.05
Duration of diabetes (months)	111.92 ± 70.17	90.16 ± 73.08	<0.05
24-hour urine protein quantification [mg/dl, M(Q1, Q3)]	2.64 (1.49,3.68)	2.40 (0.99,4.30)	
eGFR (EPI),ml/min/1.73m^2^	53.81 ± 25.99	64.39 ± 33.32	
Alanine transaminase [U/L,M(Q1,Q3)]	17.50 (12.50,26.00)	22.50 (14.00,34.00)	<0.05
Blood uric acid [μmol/L]	367.50 (338.50,467.75)	402.00 (334.25,469.00)	
Blood urea nitrogen [mmol/L,M(Q1,Q3)]	7.66 (5.97,12.41)	7.81 (5.80,9.96)	
Serum creatinine [μmol/L,M(Q1,Q3)]	122.50 (85.00,196.00)	110.00 (80.00,166.00)	
Serum albumin [g/L]	33.13 ± 5.92	34.27 ± 7.86	<0.05
Total cholesterol [mmol/L]	4.77 ± 1.41	4.55 (3.94,5.89)	
Triglycerides [mmol/L,M(Q1,Q3)]	1.64 (1.15,2.51)	1.96 (1.44,2.76)	<0.05
Low-Density lipoprotein cholesterol (LDL) [mmol/L]	3.04 ± 1.19	2.85 (2.14,3.75)	
Blood potassium [mmol/L]	4.20 ± 0.64	4.06 (3.77,4.29)	
Bilirubin [μmol/L, M(Q1,Q3)]	6.65 (5.75,9.80)	8.90 (7.07,11.35)	<0.05
HbA1c(%)	7.35 (6.18,8.53)	6.90 (6.18, 8.03)	
PLT[*10^9^/L]	208.00 (176.75,245.00)	209.50 (169.50,257.00)	
ACR[mg/mmol,M(Q1,Q3)]	1.74 (0.89,3.09)	1.16 (0.44,2.35)	<0.05
NAG [U/L,M(Q1,Q3)]	12.29 (7.80,19.10)	12.74 (9.02,20.15)	
Cystatin C [mg/L, M(Q1,Q3)]	1.40 (1.02, 2.13)	1.40 (0.99,1.83)	
DR [cases (%)]	72 (71.3)	62 (33.7)	<0.05
Hypertension [cases (%)]	83 (83)	146 (79.3)	
Cardiovascular diseases [cases (%)]	24 (24)	26 (14.1)	<0.05
Hyperthyroidism and hypothyroidism [cases (%)]	3 (3.1)	13 (7.1)	
Anaemia [cases (%)]	32 (31.7)	40 (21.7)	
Diseases of the urinary system [cases (%)]	20 (20.2)	23 (12.5)	
Hyperlipidemia [cases (%)]	30 (30.3)	58 (31.5)	
Chronic liver disease and cirrhosis [cases (%)]	16 (16)	22 (12)	
Nephrotic syndrome [cases (%)]	8 (8.1)	21 (11.4)	
Haematuria (>3HP) [cases (%)]	55 (54.5)	113 (61.4)	
Massive proteinuria (>3.5) [cases (%)]	46 (44)	71 (38.6)	
Glucocorticoids [cases (%)]	5 (5.1)	43 (23.4)	<0.05
Immunosuppressants other than hormones [cases (%)]	1 (1)	22 (12)	<0.05
TWP[cases (%)]	41 (40.6)	47 (25.5)	<0.05
Insulin and insulin analogs [cases (%)]	73 (72.3)	80 (43.5)	<0.05
α-Glucosidase Inhibitors [cases (%)]	38 (37.6)	75 (40.8)	
Meglitinides [case (%)]	8 (7.9)	38 (20.7)	<0.05
Sulfonylureas [case (%)]	6 (5.9)	11 (6)	
Biguanides [cases (%)]	17 (16.8)	38 (20.7)	
Dipeptidyl Peptidase-4 (DPP-4) Inhibitors [cases (%)]	11 (10.9)	25 (13.6)	
Sodium-Glucose Cotransporter-2 (SGLT-2) Inhibitors [cases (%)]	9 (8.9)	24 (13)	
Beta-Adrenergic blockers [cases (%)]	30 (29.7)	46 (25)	
ACEI/ARB [cases (%)]	60 (59.4)	107 (58.2)	
CCB[cases (%)]	68 (67.3)	118 (64.1)	
NSAIDs [cases (%)]	2 (2)	4 (2.2)	
Lipid-Lowering agents [cases (%)]	58 (57.4)	103 (56)	
Anticoagulants or antiplatelet drugs [cases (%)]	18 (17.8)	34 (18.5)	

### 3.2 Classification of pathology

Pathological examination of renal biopsies in the DKD+NDKD group identified a spectrum of over ten distinct pathological types, predominantly characterized by primary glomerular diseases, complemented by secondary glomerular conditions. The predominant pathological type within the DKD+NDKD group was IgA nephropathy, which constituted 37.50% of the patient population, followed by tubular interstitial disease, representing 22.80% of cases (**Table 2**). In terms of glomerular pathological scores, the DKD group exhibited a significantly higher prevalence of grade III scores, affecting 68.1% of patients, in contrast to the DKD+NDKD group, where only 26.9% of patients were affected (P < 0.05). Conversely, the DKD+NDKD group had a higher proportion of grade IIa scores, with 53.8% of patients, compared to the DKD group, where only 5.5% of patients were affected (P < 0.05) (**Table 3**). No statistically significant differences were observed between the two groups in terms of hyaline arteriolosclerosis, arteriosclerosis, and tubular atrophy (IFTA) scores (P > 0.05). However, this may also be related to insufficient sample size, which may require a larger sample size for further research. However, the DKD group showed a significantly higher proportion of tubulointerstitial inflammation scores of one point compared to the DKD+NDKD group (P < 0.05). In contrast, the proportion of patients with a tubulointerstitial inflammation score of two points was higher in the DKD+NDKD group than in the DKD group (P < 0.05).

**Table 2:** Distribution of pathological changes in patients with type 2 diabetes mellitus with combined renal lesions.

Pathological changes	DKD+NDKD group (n)
IgA nephropathy	69
Tubulointerstitial lesion	42
Sclerosing glomerulopathy	32
Membranous nephropathy	16
Proliferative glomerulopathy	11
Immune complex-mediated glomerulonephritis	4
Focal segmental glomerulosclerosis	2
Hepatitis B-associated Glomerulonephritis	2
Crescentic glomerulonephritis	2
Ischaemic kidney injury	2
Metabolism-related lesions	1
Glomerular podocytes	1
IgM-λ restricted expression	1

**Table 3:** Renal pathology assessment in patients with diabetic nephropathy.

Pathological changes	Score	All	DKD	DKD+NDKD	P
IFTA	1	26 (14.1%)	10 (11%)	16 (17.2%)	P>0.05
	2	119 (64.7%)	57 (62,6%)	62 (66.7%)	
	3	39 (21.2%)	24 (26.4)	15 (16.1%)	
Tubulointerstitial inflammation	1	130 (70.7%)	71 (78%)	59 (63.4%)	P=0.03
	2	54 (29.3%)	20 (22%)	34 (36.6%)	
Hyaline arteriolosclerosis	0	2 (1.1%)	0	2 (2.2%)	P>0.05
	1	27 (14.7%)	16 (17.6%)	11 (11.8%)	
	2	155 (84.2%)	75 (82.4%)	80 (86%)	
Arteriosclerosis	0	60 (32.6%)	29 (31.9%)	31 (33.3%)	P>0.05
	1	78 (42.4%)	45 (49.5%)	33 (35.5%)	
	2	46 (25%)	17 (18.7%)	29 (31.2%)	
Glomerular pathology grading	I	0	0	0	P<0.001
	IIa	55 (29.9%)	5 (5.5%)	50 (53.8%)	
	IIb	31 (16.8%)	15 (16.5%)	16 (17.2%)	
	III	87 (47.3%)	62 (68.1%)	25 (26.9%)	
	IV	11 (6%)	9 (9.9%)	2 (2.2%)	

### 3.3 Propensity score matching analysis

Considering the initial disparities in baseline data between the two cohorts, a 1:1 propensity score matching (PSM) approach was implemented to ensure comparability. Key variables selected for matching included BMI, diabetes duration, serum albumin, triglyceride, and the presence of cardiovascular disease. Following the PSM, the analytical dataset was refined to include 186 patients. This method effectively adjusted for the confounding effects of BMI, diabetes duration, serum albumin, triglyceride, and cardiovascular disease. After the PSM, the DKD and DKD+NDKD groups each comprised 93 cases. The post-PSM analysis revealed statistically significant differences in 24-hour urinary protein quantification, ACR, and DR (P < 0.05). Specifically, the DKD group demonstrated higher levels of 24-hour urinary protein quantification and ACR, along with a more prolonged duration of diabetic retinopathy, in comparison to the DKD+NDKD group (**Table 4**).

**Table 4:** Distribution of baseline parameters after PSM in DKD group and DKD+NDKD group.

Parametric	DKD (n=94)	DKD+NDKD (n=94)	P
Age (years)	52.68 ± 9.82	54.14 ± 10.52	
Male [cases (%)]	66 (71)	72 (77.4)	
BMI(kg/m^2^)	24.51 ± 3.14	24.31 ± 2.80	
Duration of diabetes (months)	120 (48,160)	84 (48,160)	
24-hour urine protein quantification [mg/dl, M(Q1, Q3)]	3.41 (2.06,5.44)	2.47 (1.1,4.27)	<0.05
eGFR(EPI),ml/min/1.73m^2^	50.60 (28.60,68.70)	50.40 (32.73,74.88)	
Blood uric acid [μmol/L]	387.64 ± 95.94	408.43 ± 101.00	
Blood urea nitrogen [mmol/L,M(Q1,Q3)]	8.53 (6.05,13.58)	8.64 (6.8,12.4)	
Serum creatinine [μmol/L,M(Q1,Q3)]	122.00 (86.00,193.00)	119.50 (85.30,178.25)	
Serum albumin [g/L]	32.47 ± 6.39	33.65 ± 6.14	
Total cholesterol [mmol/L]	4.83 (3.85,6.11)	4.64 (3.44,5.75)	
Triglycerides [mmol/L,M(Q1,Q3)]	1.49 (1.1,2.53)	1.62 (1.13,2.40)	
LDL [mmol/L]	3.06 (2.27,3.88)	2.83 (1.98,2.53)	
Blood potassium [mmol/L]	4.13 ± 0.54	4.17 ± 0.55	
HbA1c(%)	7.10 (6.20,8.50)	6.70 (6.00, 8.10)	
PLT[*10^9^/L]	210.00 (172.00,281.00)	204.00 (164.25,237.75)	
ACR[mg/mmol,M(Q1,Q3)]	2.49 (1.00,4.17)	1.26 (0.52,3.02)	<0.05
NAG [U/L,M(Q1,Q3)]	12.99 (9.20,19.70)	12.00 (9.12,20.71)	
Cystatin C [mg/L, M(Q1,Q3)]	1.55(1.07, 2.17)	1.43 (1.06,2.07)	
DR [cases (%)]	66 (71)	35 (37.6)	<0.05
Hypertension [cases (%)]	77 (81.7)	75 (80.6)	
Cardiovascular diseases [cases (%)]	21 (22.6)	15 (16.1)	
Hyperthyroidism and hypothyroidism [cases (%)]	4 (4.3)	5 (5.4)	
Anaemia [cases (%)]	29 (31.2)	21 (22.6)	
Diseases of the urinary system [cases (%)]	19(20.4)	14 (15.1)	
Hyperlipidemia [cases (%)]	30 (32.3)	24 (25.8)	
Chronic liver disease and cirrhosis [cases (%)]	15 (16.1)	14 (15.1)	
Nephrotic syndrome [cases (%)]	8 (8.6)	8 (8.6)	
Haematuria (>3HP) [cases (%)]	52 (55.9)	57 (57)	
Massive proteinuria (>3.5) [cases (%)]	42 (45.2)	32 (34.4)	
Glucocorticoids [cases (%)]	5 (5.4)	23 (24.7)	<0.05
Immunosuppressants other than hormones [cases (%)]	1 (1.1)	9 (9.7)	<0.05
TWP[cases (%)]	39 (41.9)	25 (25.9)	<0.05
Insulin and insulin analogues [cases (%)]	69 (74.2)	47 (50.5)	<0.05
α-Glucosidase Inhibitors [cases (%)]	36 (38.7)	43 (46.2)	
Sodium-Glucose Cotransporter-2 (SGLT-2) Inhibitors [cases (%)]	6 (6.5)	12 (12.9)	
Beta-Adrenergic blockers [cases (%)]	29 (31.2)	22 (23.7)	
ACEI/ARB[cases(%)]	55 (59.1)	52 (55.9)	
CCB[cases(%)]	64 (68.8)	59 (63.4)	
Anticoagulants or antiplatelet drugs [cases (%)]	17 (18.3)	19 (20.4)	

### 3.4 Analysis of survival

**Figure 1:**
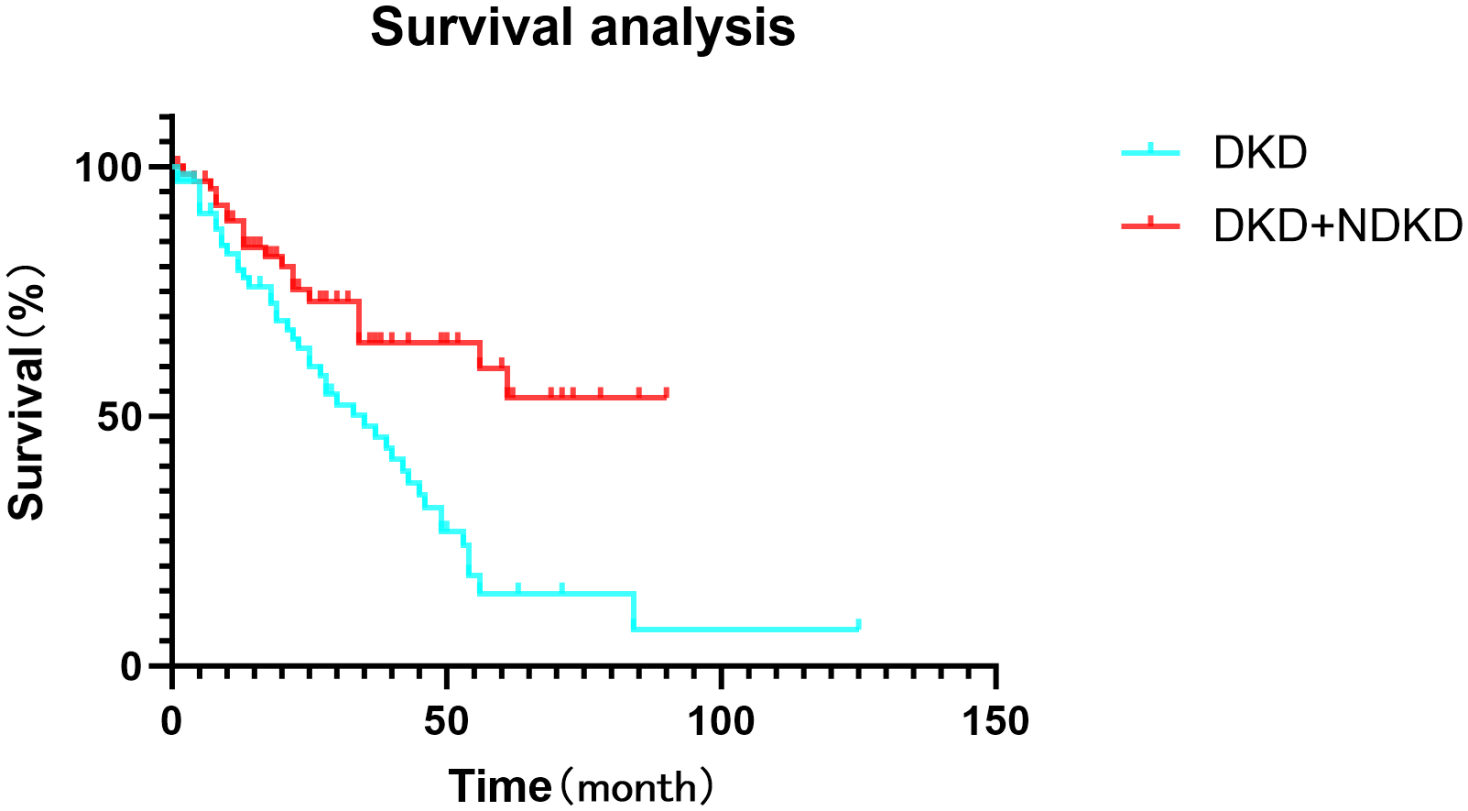
Analysis of survival for DKD and DKD+NDKD group at 20, 40, and 60 months.

Following propensity score matching, we evaluated the incidence of endpoint events, defined as end-stage renal disease, initiation of renal replacement therapy, or a continuous decline in estimated glomerular filtration rate (eGFR) exceeding 50%, in both the DKD and DKD+NDKD groups. In the DKD group, 44 cases (66.7%) experienced endpoint events, whereas in the DKD+NDKD group, 24 cases (32.9%) reached these endpoints (P < 0.05). Cumulative survival curve analysis indicated that the incidence of endpoint events was 21.2% and 15.1% at 20 months, 40.9% and 24.66% at 40 months, and 63.6% and 30.1% at 60 months for the DKD and DKD+NDKD groups, respectively. Patients in the DKD group exhibited a higher rate of endpoint events at 20, 40, and 60 months compared to those in the DKD+NDKD group (all P < 0.05) (**Figure 1**).

### 3.5 Univariate and multivariate COX regression analysis for DKD+NDKD

To identify prognostic factors for DKD and DKD+NDKD, we conducted a Cox regression analysis with endpoint events as the dependent variables. The univariate Cox regression analysis indicated that in the DKD+NDKD group, serum creatinine, 24-hour urinary protein quantification, eGFR, serum albumin, urea nitrogen,ACR, NAG, cystatin C, and massive proteinuria (>3.5 g/day) were associated with adverse renal outcomes. However, age, gender, diabetes duration, BMI, serum uric acid, HbA1c, hematuria, LDL, nephrotic syndrome, and DR were not significantly associated with adverse renal outcomes. Subsequently, a multivariate Cox regression analysis was performed on the clinically significant indicators (P < 0.05) identified from the univariate analysis. This analysis revealed that cystatin C [hazard ratio (HR) 2.688, 95% confidence interval (CI) 1.035-6.879] is an independent prognostic risk factor for patients with DKD+NDKD (**Table 5**).

**Table 5:** Cox regression analysis of prognosis in patients with DKD+NDKD.

Factors	Univariate analysis			Multivariate analysis		
	EXP(B)	HR (95%CI)	P	EXP(B)	HR (95%CI)	P
Age	0.975	0.941-1.012	0.18			
Gender	0.441	0.188-1.033	0.059			
Duration of Diabetes	0.998	0.992-1.003	0.454			
BMI	0.920	0.793-1.066	0.268			
GFR	0.977	0.960-0.994	0.007	1.015	0.992-1.038	0.196
24-hour Urinary Protein	1.138	1.036-1.251	0.007	0.862	0.607-1.223	0.405
Serum Uric Acid	1.001	0.996-1.005	0.758			
Scr	1.004	1.002-1.006	<0.001	0.995	0.983-1.006	0.338
Alb	0.904	0.847-0.965	0.002	0.991	0.887-1.107	0.873
BUN	1.119	1.063-1.179	<0.001	1.197	0.959-1.495	0.112
HbA1c	1.002	0.774-1.298	0.986			
Hematuria	1.366	0.583-3.201	0.473			
LDL	1.071	0.788-1.456	0.661			
ACR	1.368	1.161-1.162	<0.001	1.271	0.835-1.936	0.263
NAG	1.042	1.004-1.081	0.03	1.048	0.997-1.102	0.066
Cystatin C	2.192	1.507-3.189	<0.001	2.668	1.035-6.879	0.042
Heavy Proteinuria (>3.5g)	0.2875	1.275-6.485	0.011	2.459	0.430-14.072	0.312
Nephrotic Syndrome	0.293	0.082-1.045	0.058			
DR	1.646	0.732-3.700	0.228			

## 4 Discussion

T2DM patients frequently exhibit comorbid DKD and NDKD, which necessitate distinct therapeutic approaches and prognostic considerations (12). Notably, the presence of CKD in diabetic patients does not invariably indicate renal impairment attributable to diabetes mellitus. Therefore, a precise differential diagnosis is crucial for tailoring treatment strategies and optimizing prognosis in T2DM patients with renal damage. Renal biopsy remains the most reliable method for ascertaining the nature of renal pathology in diabetic patients (10). However, given its invasive nature, renal biopsy is not without limitations (13–15). Current clinical guidelines worldwide do not uniformly define the indications for renal biopsy in this patient population, underscoring the need to elucidate further the predictive value of laboratory indices and clinical features for pathological classifications, thereby assessing the necessity of renal biopsy.

Our study encompassed 285 patients with T2DM combined with CKD who were treated at Hangzhou Hospital of Traditional Chinese Medicine and diagnosed by renal biopsy for a period of 11 years from January 2012 to February 2023. Patients were classified into two groups based on renal biopsy findings: those with isolated DKD and those with DKD+NDKD. Statistical analysis, conducted using SPSS 25.0, revealed significant differences between the groups. The study aimed to delve deeper into the clinical predictors and prognosis of DKD+NDKD and to scrutinize the indications for renal biopsy in T2DM patients with CKD. Accurate diagnosis and timely treatment are essential to retard the progression of kidney disease, enhance long-term patient prognosis, and mitigate the incidence of end-stage renal disease (ESRD) in in patients with DKD+NDKD.

According to Chinese literature, the detection rate of DKD+NDKD in diabetic patients with CKD ranges from 25.7% to 31.44% (16), with IgA nephropathy and membranous nephropathy (MN) being common pathological types. Variations in the proportion of different pathological types may be attributed to differences in the age and ethnicity of the studied patients. Some studies suggest that the predominant pathological type in the DKD+NDKD group is DKD associated with hypertensive nephropathy (17), while others propose that DKD with MN and DKD with IgA nephropathy are more common (18). In our study, IgA nephropathy was the primary pathological change in the DKD+NDKD group, accounting for 37.5% of the patients, followed by tubulointerstitial lesions, which affected 22.8% of the patients. By evaluating the patients’ glomerular pathological grading, we found that the glomerular grading in the DKD+NDKD group was mostly grade IIa, while the DKD group had a higher proportion of grades III and IV (P < 0.05), which may be due to the lack of timely diagnosis of patients with DKD due to the lack of clinical symptoms in the early stage of the disease. Therefore, it is necessary to carry out regular urinalysis and screening for diabetic patients to achieve early diagnosis and treatment.

The pathogenesis of DKD is highly complex. A synthesis of existing knowledge indicates that diabetic patients are prone to renal microvascular disease (19–21). The severity and duration of hyperglycemia are correlated with microvascular complications in diabetic patients. DR, a prevalent microvascular disease in diabetic patients, serves as a risk indicator for systemic vascular complications (22). Numerous studies have identified that brief diabetes duration and the absence of DR can predict NDKD, whereas the presence of DR significantly predicts DKD (23–25). A cohort study indicated that diabetic nephropathy independently increases the risk of developing and progressing diabetic retinopathy (26). Our study employed a 1:1 propensity score matching to minimize the influence of confounding factors between the DKD and DKD+NDKD groups. It was observed that the diabetes duration in the DKD+NDKD group was significantly shorter than that in the DKD group, with a median diabetes duration of over ten years in the DKD group and under seven years in the DKD+NDKD group. The incidence of DR was 71% in the DKD alone group and 37.6% in DKD+NDKD group. Thus, the utility of DR as an indicator for renal biopsy to reduce the misdiagnosis rate of DKD+NDKD warrants further investigation.

Some studies have suggested that elevated triglyceride levels have a specific predictive effect on the occurrence of NDKD (27). Our study found that triglyceride levels were significantly higher in DKD+NDKD group compared to the simple DKD group, leading to the speculation that higher triglyceride levels could predict the occurrence of DKD+NDKD. However, the correlation between triglyceride levels and the occurrence of DKD+NDKD remains a subject that requires further exploration, as many studies have not found such a link.

Previous studies have identified that hematuria has a specific predictive effect on NDKD, with the presence of dysmorphic red blood cells potentially offering greater predictive value for NDKD (28). Hematuria in DKD patients may be associated with changes in glomerular hemodynamics, increased pressure, or aneurysm rupture, potentially leading to non-glomerular hematuria (29). NDKD typically presents with glomerular hematuria. Our study found no statistically significant difference in the incidence of hematuria between the two groups. However, due to the limited number of patients who underwent hematuria localization examination, statistical analysis of this parameter was not feasible. Therefore, further investigation of the morphology of red blood cells in hematuria among patients with isolated DKD and DKD+NDKD is warranted. Moreover, studies have shown that hematuria is not uncommon in DKD patients, and its predictive value for NDKD is not as robust as anticipated (30). Liu et al. reported that the incidence of microscopic hematuria in T2DM patients diagnosed via renal biopsy pathology increased from 16.7% (1993-2003) to 32.3% (2004-2012) (31). Akimoto et al. identified a significantly higher incidence of microscopic hematuria in DKD patients with nephrotic syndrome or renal dysfunction (32). Thus, hematuria as a basis for identification has significant limitations and necessitates further exploration.

In our study, ESRD served as the endpoint. We found that DKD patients diagnosed through renal biopsy had a relatively poor prognosis, with a 5-year renal survival rate of about 40% (33). Interestingly, those with both DKD and NDKD had a better survival rate than those with only DKD. DKD patients often have a longer history of diabetes, worse renal function, and more complications, which may be due to limited treatment options. In contrast, patients with DKD+NDKD having a better renal prognosis may be associated with clinical stabilization or remission of certain diseases, such as IgAN and MN, which may be treatable by RAAS inhibitors or immunosuppressive therapy, and therefore the patient’s renal function is restored and preserved. This suggests that in clinical practice, it is still necessary to actively perform renal biopsy in patients considered for DKD to clarify whether the patient has DKD+NDKD, which can effectively help some patients with combined NDKD to better delay renal failure and improve prognosis. Univariate analysis highlighted several prognostic risk factors, including serum creatinine, eGFR, serum albumin, hemoglobin, urea nitrogen, ACR, Cystatin C, and significant proteinuria (>3.5g). This suggests that in patients with type 2 diabetes mellitus who are unable to undergo renal biopsy, keeping an eye on the relevant indicators can be helpful in the clinical diagnosis of DKD+NDKD. The comprehensive multivariate Cox regression analysis indicated that cystatin C, characterized by a hazard ratio of 2.688 (with a 95% confidence interval ranging from 1.035 to 6.879), stands out as a significant independent predictor of adverse outcomes for patients dealing with both DKD+NDKD. Cystatin C, a cysteine protease inhibitor, regulating the activity of cathepsins S and K, have multiple functions in human vascular pathophysiology (34). Cystatin C, which is cleared only by glomerular filtration, is an endogenous marker reflecting changes in glomerular filtration rate, and it is essential to evaluate the renal function status of patients with DKD and DKD+NDKD with a reliable GFR in the early stages of the disease, and several studies have found that the level of cystatin C is elevated earlier than creatinine (35, 36). Clinical assessment of GFR by testing blood creatinine lacks sufficient sensitivity for mild renal injury, whereas Cystatin C can respond sensitively to mild renal injury, and regular testing of Cystatin C in patients with DKD and DKD+NDKD allows for dynamic observation of disease progression. In conclusion, cystatin C is a new endogenous marker reflecting GFR, which is a sensitive and accurate indicator of early renal impairment.

This study has limitations, including its single-center, retrospective design over an extended period. There are variations in the recommended renal biopsy indications across different periods, inevitably leading to selective bias. Secondly, the overall sample size of this study is small, which could be increased to enhance the statistical power. Thirdly, the absence of uniform renal biopsy indicators and diagnostic criteria for DKD in T2DM patients may introduce bias into the research findings. Further large-sample, prospective, multi-center clinical research is warranted to explore patients’ pathogenesis and prognostic factors.

## 5 Conclusions

The coexistence of DKD alongside NDKD is a frequently observed issue among individuals with diabetes. Our research has pinpointed various risk factors that are linked to poor kidney outcomes in diabetic patients who also suffer from NDKD. These factors include high levels of serum creatinine, a decrease in glomerular filtration rate (GFR), low serum albumin levels, increased urea nitrogen, a raised ACR, enhanced activity of the NAG, elevated cystatin C levels, and the occurrence of substantial proteinuria exceeding 3.5 g per day. Among them, cystatin C is a significant independent predictor of poor prognosis in patients with DKD+NDKD. However, considering the study was conducted at a single center, it is imperative that these findings undergo further scrutiny and verification across various centers throughout China to ascertain their broader applicability.

## Data Availability

The raw data supporting the conclusions of this article will be made available by the authors, without undue reservation.
